# Promoting healthy behaviours – do we need to practice what we preach?

**DOI:** 10.1080/17571472.2015.1113716

**Published:** 2015-12-24

**Authors:** Alison E. While

**Affiliations:** ^a^ Florence Nightingale Faculty of Nursing and Midwifery, King’s College London, London, UK

**Keywords:** Public health, doctors, nurses, obesity, physical activity, smoking, alcohol, health behaviours, behaviour change

## Abstract

The UK faces a public health challenge arising from unhealthy behaviours. Some health care workers engage in the same unhealthy behaviours as the general population. This paper explores the issues arising from some primary care staff adopting unhealthy behaviours upon healthcare organisations, professional practice and patient perceptions in terms of the promotion of health in the primary care setting.

## Why it matters to me

The primary care workforce is at the frontline for promoting healthy behaviours but the social judgements of their patients may undermine the credibility of health professionals as health promoters.

## Key messages

• The health care workforce exhibits the same health behaviours as the general population.
• Unhealthy behaviours increase ill health and unplanned staff absences which impact negatively upon care delivery.
• Personal health behaviours of health professionals may influence their clinical practice.
• Health professionals with unhealthy behaviours may be less credible as health promoters.
• Primary care staff are the face of the NHS and can be role models for their patients.

## Background

The Chief Medical Officer’s [1] (CMOs) report sets out the public health challenge of the increasing levels of obesity across the population and the associated increased risk of cardiovascular disease, diabetes and some cancers. While the causes of obesity are multi-factorial and include increased sugar consumption, the CMO also notes the increase in sedentary lifestyles. The report also highlights the increase in liver disease mortality reflecting the extent to which some drink alcohol to excess and its normalisation within popular culture together with its relatively cheap cost. Tobacco use also continues as an avoidable risk factor of cancer, and cardiovascular and respiratory diseases with people in more deprived areas disproportionately smoking cigarettes compared to their more affluent counterparts.[1]

The concerns raised by the CMOs report have been reiterated in the future strategy for the NHS [2] which calls for a ‘radical upgrade in prevention and public health’ with a particular focus on obesity, smoking and alcohol use alongside the implementation of integrated care to make the NHS sustainable. The centrality of primary care within health care delivery is recognised as is its role to promote healthy lifestyles both within health assessments and the on-going care of patients with long term conditions. Within many practices, nurses and other staff are conducting the NHS health checks and monitoring patients with long-term conditions with general practitioners focusing their efforts upon the clinically unstable and acutely ill in attempt to use the limited skill resources most efficiently.

The evidence suggests that the health care workforce exhibits the same health behaviours as the general population.[3] Thus the Government estimated that 300,000 NHS staff were obese, with a further 400,000 overweight in 2008 [3] and these figures are likely to have risen in line with the population trends. But as with these body weight data, there are no data specifically relating to the primary health care workforce regarding their smoking or alcohol use. Indeed, there is every reason to expect that they are the same as the general population including the socio-economic differences across the professional and support workforces.

Nonetheless primary care staff, like all healthcare staff, are expected to exhibit professional behaviours at all times regardless of their personal health behaviours. This means that every opportunity should be taken to promote health [4] and optimal self-management to reduce the use of health services.[5]

## Question

Do the health behaviours of health professionals matter?

## Impact upon healthcare organisations

At the organisational level, services depend upon motivated staff capable of delivering high-quality care to their patients and this is undermined when there are staff absences which reduce care capacity and disrupt continuity of care.[6] Unplanned staff absence also adds to the cost of care delivery when services become less efficient and agency staff are employed to cover essential personnel.[6] Staff absence also creates workload stress to those who remain and may be the cause of work-related ill health. Indeed, health professionals generally have higher rates of mental ill health than the general population.[6] Further, patient safety may be compromised by both understaffing and by underperforming healthcare teams as they struggle to deliver care as was all too evident in Francis Inquiry.[7]

## Impact upon professional practice

There is a growing body of evidence that the personal health behaviours of health professionals may influence how they practice clinically. Two systematic reviews suggest that the personal body weight of doctors and nurses are related to: (1) their attitudes towards weight management [8]; and (2) their actual weight management practices [9] as health professionals. Further, there is increasing evidence that an obesogenic society is normalising larger body weight sizes with Zhu et al. [10] reporting that a quarter of student nurses (*n* = 355) and a third of qualified nurses (*n* = 409) in their sample misperceived their weight status in favour of a lower BMI which may impact upon their assessment of patients in need of weight management support. Further, although the nurses who worked in the community reported more weight management practices, Zhu et al.’s [11] path analysis revealed that the self-efficacy [12] of the qualified nurses (*n* = 420) directly and positively predicted their weight management practices; that is, the nurse’s belief in their ability to deliver weight management practices is important to understand their professional practice. This suggests that the enhancement of nurses’ self-efficacy may be an effective strategy to improve their weight management practices.

Similarly, personal engagement in physical activity appears to be related to levels of physical activity promotion although the self-report measurement of physical activity is problematic.[13] A study [14] of qualified nurses (*n* = 623) has reported that many nurses are not achieving the recommended levels of physical activity. Further, 25% of the sample were at risk of hazardous drinking or had an active alcohol disorder and 11% were current smokers and 17% were past smokers. This study confirmed a relationship between the nurses’ personal health behaviours and their physical activity health promotion practices. Two other systematic reviews suggest that personal tobacco use [15] and alcohol consumption [16] are also related to health promotion practices.

## Patient perceptions

The personal health behaviours of health professionals may influence how patients view their credibility as a health promoter. Indeed, negative attitudes towards obese people are widespread across society [17] with similar attitudes towards obese doctors [18] with patients reporting less likelihood to trust or follow their medical advice. A small qualitative study [19] with 21 overweight/obese adults suggests that health professionals who model healthy lifestyles are perceived as more credible and more motivating in their health promotion efforts. This may be important for supporting patients to achieve desired health behaviour change.

## Conclusions

The health care workforce, including those working in primary care, is likely to exhibit the same health behaviour trends as the general population. This may have important implications for health care delivery both in terms of staff absences and suboptimal health promotion. Perhaps we need to consider what we can reasonably expect of primary healthcare staff as role models and as the face of the NHS. A good first step may be to implement NICE guidelines relating to the promotion of health and well-being within primary care workplace settings thereby supporting staff in their adoption of healthy lifestyles. The relevant NICE guidelines include those relating to promoting physical activity,[20,21] mental well-being,[22] smoking cessation [23] and weight management.[24] As the first point of contact primary care in London has much to contribute to public health.[25]

### Ethical approval

None required
